# Differential effect of lactate on synovial fibroblast and macrophage effector functions

**DOI:** 10.3389/fimmu.2023.1183825

**Published:** 2023-05-22

**Authors:** Valentina Pucino, Meriam Nefla, Vincent Gauthier, Ghada Alsaleh, Sally A. Clayton, Jennifer Marshall, Andrew Filer, Andy R. Clark, Karim Raza, Christopher D. Buckley

**Affiliations:** ^1^ Rheumatology Research Group, Institute of Inflammation and Ageing, University of Birmingham, Birmingham, United Kingdom; ^2^ Kennedy Institute of Rheumatology, Oxford University, Oxford, United Kingdom; ^3^ Sandwell and West Birmingham National Health System (NHS) Trust, Birmingham, United Kingdom

**Keywords:** lactate, fibroblasts, macrophages, arthritis, cell metabolism

## Abstract

**Introduction:**

The synovial membrane is the main site of inflammation in rheumatoid arthritis (RA). Here several subsets of fibroblasts and macrophages, with distinct effector functions, have been recently identified. The RA synovium is hypoxic and acidic, with increased levels of lactate as a result of inflammation. We investigated how lactate regulates fibroblast and macrophage movement, IL-6 secretion and metabolism via specific lactate transporters.

**Methods:**

Synovial tissues were taken from patients undergoing joint replacement surgery and fulfilling the 2010 ACR/EULAR RA criteria. Patients with no evidence of degenerative or inflammatory disease were used as control. Expression of the lactate transporters SLC16A1 and SLC16A3 on fibroblasts and macrophages was assessed by immunofluorescence staining and confocal microscopy. To test the effect of lactate in vitro we used RA synovial fibroblasts and monocyte-derived macrophages. Migration was assessed via scratch test assays or using trans-well inserts. Metabolic pathways were analysed by Seahorse analyser. IL-6 secretion was determined by ELISA. Bioinformatic analysis was performed on publicly available single cell and bulk RNA sequencing datasets.

**Results:**

We show that: i) SLC16A1 and SLC16A3 which regulate lactate intake and export respectively, are both expressed in RA synovial tissue and are upregulated upon inflammation. SLC16A3 is more highly expressed by macrophages, while SLC16A1 was expressed by both cell types. ii) This expression is maintained in distinct synovial compartments at mRNA and protein level. iii) Lactate, at the concentration found in RA joints (10 mM), has opposite effects on the effector functions of these two cell types. In fibroblasts, lactate promotes cell migration, IL-6 production and increases glycolysis. In contrast macrophages respond to increases in lactate by reducing glycolysis, migration, and IL-6 secretion.

**Discussion:**

In this study, we provide the first evidence of distinct functions of fibroblasts and macrophages in presence of high lactate levels, opening new insights in understanding the pathogenesis of RA and offering novel potential therapeutic targets.

## 1 Introduction

Rheumatoid arthritis (RA) is a systemic immune-mediated disease that manifests principally as inflammation in synovial joints. Despite the development of game changing biologic therapies, few people with RA achieve a state of durable drug-free disease remission. Consequently, the disease is associated with high personal, societal and economic costs. Therefore, innovations are needed to better understand the mechanisms regulating disease initiation and persistence allowing the identification of potential new treatments, able to suppress inflammation and induce tissue repair. We have recently identified anatomically distinct subsets of synovial fibroblasts with non-overlapping effector functions during inflammatory arthritis (1–3). Sub lining layer fibroblasts (CD90^+^ in human) drive inflammation whilst those in the lining layer (CD90^-^) promote cartilage damage and bone remodelling (1–3). These findings are complemented by the discovery of distinct subsets of synovial macrophages (4, 5). Epithelial-like CX_3_CR1^pos^ lining layer macrophages (equivalent to MerTK^+^ macrophages in human, 5) restrict the inflammatory reaction triggered in the sub-lining by providing an anti-inflammatory shield for intra-articular structures (4, 5). MerTK is indeed highly expressed by anti-inflammatory regulatory macrophages (5, 6) while its expression is low in pro-inflammatory macrophages (7). In normal joints, these barrier forming macrophages maintain their numbers through a pool of locally renewing CX_3_CR1^neg^ (MerTK^neg^) macrophages that reside in the sub lining layer, differentiate into CX_3_CR1^pos^ (MerTK^pos^) macrophages, and migrate to the lining layer (4, 5). In active RA, these precursors are replaced by inflammatory monocyte-derived macrophages which are retained in the sub lining layer and are unable to replenish the lining layer pool leading to barrier layer disruption (4, 5). Fibroblasts and macrophages both acquire a pro-inflammatory and destructive phenotype during RA (1–5). In addition, recent evidence has established macrophage-fibroblast crosstalk as pivotal for the maintenance of synovial tissue homeostasis and the failure of this cellular interchange leads to the persistence of joint inflammation and disease progression (5).

At inflammatory sites (e.g. RA synovitis) energy sources become limiting and inflammatory cells adapt metabolically in order to perform their homeostatic functions (8).

The pro-inflammatory phenotype of synovial fibroblasts is characterised by a metabolic shift toward glycolysis, which is accompanied by upregulation of glycolytic enzymes such as Pyruvate kinase (PKM2), GLUT1, hexokinase 2 and 6-Phosphofructo-2-Kinase/Fructose-2,6-Biphosphatase 3 (PFKFB3) (9). We have recently demonstrated that synovial fibroblasts isolated from RA patients display a mitochondrial dysfunction in response to TNFα (10). Similarly, pro-inflammatory subsets of synovial macrophages that are expanded in active RA display elevated expression of glycolytic genes (11). Circulating monocytes from RA patients were found with increases in both glycolytic and mitochondrial metabolism relative to healthy controls (12–14). This high glycolytic state is accompanied by increased expression of glycolytic enzymes (e.g. HK2, PFKFB3) as consequence of hypoxia inducible factor 1 alpha (HIF1α) activation (13). Mitochondrial dysfunction is associated with enhanced mitochondrial respiration, biogenesis, and alterations in mitochondrial morphology (13, 14). A similar metabolic phenotype was identified in CD14^+^ monocytes isolated from subject with arthralgia at risk of RA (13). Interestingly blockade of STAT3 signalling reversed this altered bioenergetic state resulting in metabolic reprogramming and resolution of inflammation (13). Lactate transporter expression in RA monocytes has not been investigated so far and little is known about metabolic alterations of synovial resident macrophages during homeostatic and disease states.

A characteristic feature of tumour and inflammatory tissues is the build-up of lactate as consequence of hypoxia and increased glycolysis and glucose uptake (15). Lactate levels reach 10 mM in inflammatory (e.g. RA synovium, 16, 17) and 30–40 mM in cancerous tissues (16). Here, lactate acts as immunomodulatory molecule regulating infiltrating immune and tissue-resident cell functions (16, 17). In addition, lactate shuttles between producer (glycolytic) and consumer (oxidative) cells fuelling their metabolism (18). This phenomenon termed the ‘lactate shuttle’ is seen in health, in cancer and inflammation (18). How lactate regulates fibroblast and macrophage interaction, and functions has not so far been investigated in arthritis and this was the aim of our study.

## 2 Methods

### 2.1 Patients

Synovial tissue samples were obtained from patients from our BEACON cohort undergoing joint replacement surgery or ultrasound-guided synovial biopsy after written and informed consent as previously reported (10, 19). All patients were naïve to treatment at the time of synovial biopsy. Diagnosis of RA was made according to the 2010 ACR/EULAR criteria (20). Patients, sex and age matched, with no clinical and histological evidence of inflammatory disease who underwent exploratory conventional arthroscopy for knee pain were used as control. Patients with concomitant metabolic, inflammatory, and neoplastic comorbidities were not included in the study. The study was approved by local ethics committee and conducted in agreement with the Declaration of Helsinki.

### 2.2 Cell culture

Fibroblasts were isolated as previously described (10). Fibroblasts were grown in media containing 10% foetal bovine serum (FBS), sodium 0.87 mM orthopyruvate, 0.87x MEM Non-essential amino acids, 1.75 mM glutamine, 87 U/ml penicillin and 87 ug/ml streptomycin. After 3-4 passages and reaching 80-90% confluence, fibroblasts were detached and used for specific experiments. All cells were at the same passage number for all experiments. Human monocytes from healthy blood donors were obtained from blood cones supplied by the National Blood and Transplant Service (ethical approval ERN_16-0191). Monocytes were isolated by negative selection using RosetteSep Human Monocyte Enrichment Cocktail (STEMCELL Technologies). Cells were then differentiated for 7 days in RPMI 1640 (Thermo Fisher Scientific) supplemented with 5% heat-inactivated FBS in presence of recombinant macrophage colony-stimulating factor (M-CSF) (50 ng/ml; PeproTech) and then used for *in vitro* experiments.

### 2.3 Immunofluorescence

After antigen retrieval step (45 minutes) and block of non-specific binding (1 hour), paraffin-embedded tissue sections were incubated at 4°C (overnight or for 1hour) with rabbit polyclonal anti-SLC16A1 Ab (1:300, Bethyl), mouse monoclonal anti-SLC16A3 (1:100, Santa Cruz), polyclonal sheep anti-CD90 (1:100, R&D), biotin anti-CD68 (1:100, Novus), goat anti-TREM2 (1:100, Abcam), mouse anti-PRG4 (1:100, Novus), and rabbit anti-CLIC5 (1:100, ThermoFisher). Streptavidin Alexa Fluor™ 594, donkey anti-Sheep IgG Alexa Fluor™ 546, donkey anti-Rabbit IgG Alexa Fluor™ 488, donkey anti-goat IgG Alexa Fluor™ 546, donkey anti-Mouse IgG Alexa Fluor™ 647 were used as secondary antibodies (1:300). Hoechst was used for nuclear staining. Slides were mounted with Prolong Gold Antifade reagent (Invitrogen). Images were acquired on confocal microscope (Zeiss LSM 780). Colocalization was measured by calculating the Pearson’s correlation coefficient using ImageJ software as described (21).

### 2.4 Migration assays

For the scratch test assay 3×10^4^ RA fibroblasts were seeded in each well of a 24 well plate in culture medium supplemented with 10% FBS. Cells were kept at 37°C and 5% CO2 for 24 hours to allow cell adhesion and to reach a confluent monolayer. Two hours before the assay the medium was replaced with a fresh 1% FBS culture medium. The confluent monolayer was then scratched with a sterile pipette tip creating a gap of ∼0.5 mm in width. We then collected digitized images at 24h and 48h and monitored the scratched area until complete closure. The distance between each extremity of the wound was measured using ImageJ software and expressed as percentage of area closure (Time 0%). Chemokinesis assays were performed using 8um trans-well inserts. One hour prior the assay, macrophages, cultured as described in 2.2, were treated with sodium lactate (10 mM) or RA fibroblast conditioned medium (10%), with or without phloretin (41 uM, Sigma). Macrophages (2x10^5^/well) were seeded in the upper trans-well chamber in RPMI supplemented with 1% FBS. The chemokine CCL7 (300 ng/ml) was added to the lower chamber. After 4 hours migrated cells in the lower chamber were manually counted choosing 3 random fields for each well using a light microscope at 40× magnification. We then calculated the percentage of migrated cells compared to control (condition without CCL7).

### 2.5 Bioenergetics

Fibroblasts and macrophages were treated or not with lactate (10 mM) for 24 hours prior the assay (day 0). On day 1 cells were seeded at concentration of 2x10^4^/well (fibroblasts) and 2x10^5^/well (macrophages) in 96 Seahorse XF cell culture plates for other 24 hours. On day 2, cells were left to equilibrate in a non-CO2 incubator at 37°C for 1 hour. Oxygen consumption rate (OCR) and extracellular acidification rate (ECAR) were analysed using Seahorse XFe96 extracellular flux analyser according to the manufacturer’s instructions. ECAR was measured in XF media in basal condition and in response to different compounds: glucose (10 mmol/L), oligomycin (2 μmol/L) and 2-Deoxyglucose (2DG, 50 mmol/L). OCR was measured in XF media supplemented with 10 mmol/L glucose, 2 mmol/L l-glutamine, and 1 mmol/L sodium pyruvate, under basal conditions and in response to oligomycin (2 μmol/L), carbonylcyanide-4-(trifluoromethoxy)-phenylhydrazone (FCCP, 5 μmol/L) and antimycin and rotenone (3 μmol/L each). All compounds were purchased from Sigma Aldrich. At least three technical replicates were used for each condition.

### 2.6 Elisa

IL-6 secreted in the supernatants of fibroblast and macrophage cultures (3x10^4^ fibroblasts/well or 2x10^5^ macrophages/well) was measured with IL-6 human ELISA kit (ThermoFisher) according to the manufacturer’s instructions.

### 2.7 Statistical analysis

Statistical significance was determined by Student’s *t* test (comparison between two groups) or ANOVA (comparison between three or more groups) using GraphPad Prism7 software. Data are expressed as mean ± SEM. Significant differences are indicated as: *p < 0.05; **p < 0.03; ***p < 0.01.

## 3 Results

### 3.1 Lactate transporter expression in synovial tissue

**Figure 1:**
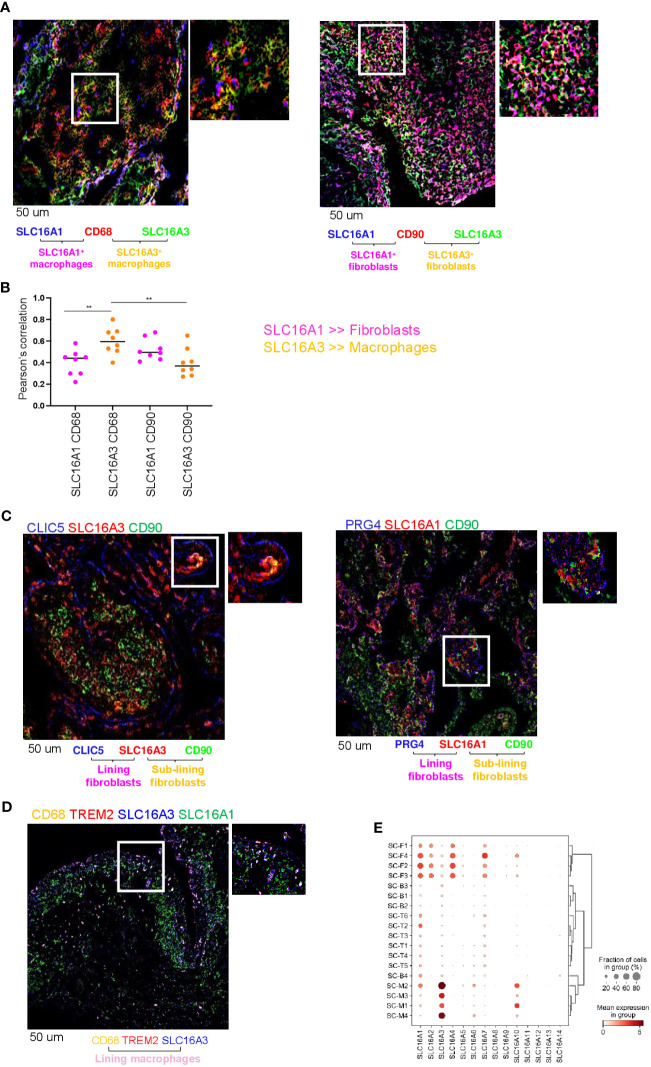
Lactate transporter expression in synovial compartment. **(A)** Representative IF staining of SLC16A1 (blue) and SLC16A3 (green) expression by synovial fibroblasts (CD90, red) and macrophages (CD68, red) from n=8 RA synovial tissues. **(B)** Quantification of the colocalization (Pearson’s correlation coefficient, ref 21) between SLC16A1 (blue) and SLC16A3 (green) and CD90 (red) and CD68 (red) within n=8 RA synovial tissues. Data are expressed as mean ± SEM. Anova test **p < 0.05; **(C)** SLC16A1 and SLC16A3 expression (red) by sub lining (CD90, green) and lining (CLIC5 or PRG4, blue) fibroblasts. **(D)** SLC16A1 and SLC16A3 expression (blue and green respectively) by sub lining (CD68^+^TREM2^-^, orange) and lining (CD68^+^TREM2^+^) macrophages. Scale bar 50 um. **(E)** scRNAseq expression of lactate transporters in synovial cellular subsets: Fibroblast: sub-lining SC-F1 (CD34^+^CD90^+^), SC-F2 (HLA-DRA^high^CD90^+^), SC-F3 (DKK^+^CD90^+^) and lining SC-F4 (CD55^+^CD90^-^PRG4^+^CLIC5^+^) subsets (ref 1, 2). Macrophages: sub-lining MerTK^neg^TREM2^neg^ SC-M1 (IL-1b^+^CD14^+^), SC-M4 (IFN-activated), and lining MerTK^pos^TREM2^pos^ SC-M2 (NUPR1^+^CD14^+^), SC-M3 (C1QA^+^CD14^+^) subsets (ref 2, 5). T cells: SC-T1 (CCR7^+^), SC-T2 (Treg cells), SC-T3 (Follicular helper T cells), SC-T4 (Granzyme K^+^), SC-T5 (Granulysin^+^, Granzyme B^+^), SC-T6 (Granzyme K^+^, Granzyme B^+^). B cells: SC-B1 (Naïve), SC-B2 (Memory), SC-B3 (Autoimmune), SC-B4 (Plasmablasts) (ref 2). Synovial tissues were taken by RA (n=36) and OA (n=15) patients (AMP dataset,ref 1, 2).

We have previously shown that RA synovial fluid contains elevated levels of lactate compared with non-inflammatory types of arthritis (e.g., osteoarthritis [OA], 17). We therefore investigated the expression and cellular localization in RA synovial tissue of two lactate transporters SLC16A1 and SLC16A3, which regulate lactate uptake and release from cells respectively (22). We performed double immunofluorescence for SLC16A1 and SLC16A3 in fibroblasts (CD90) or macrophages (CD68). We found that both transporters are expressed in RA synovial tissue (**Figure 1A, B**). CD68^+^ macrophages preferentially expressed SLC16A3 (lactate exporter), whilst SLC16A1 (lactate importer) was similarly expressed by both cell types (**Figures 1A, B**). SLC16A1 expression by fibroblasts was higher than SLC16A3 however this did not reach statistical significance (**Figures 1A, B**). Confocal images of RA synovial compartments showed that SLC16A1 and SLC16A3 were expressed by both sub-lining (CD90) and lining (CLIC5 and PRG4) fibroblast subsets, while SLC16A3 was mainly expressed by CD68^+^TREM2^+^ lining macrophages (**Figures 1C, D**). These expression patterns were confirmed in an independent publicly available single-cell RNA sequencing dataset obtained from synovial tissues of RA and OA patients (1, 2) (see figure legend, **Figure 1E**).

### 3.2 Lactate regulates fibroblast and macrophage metabolism

**Figure 2:**
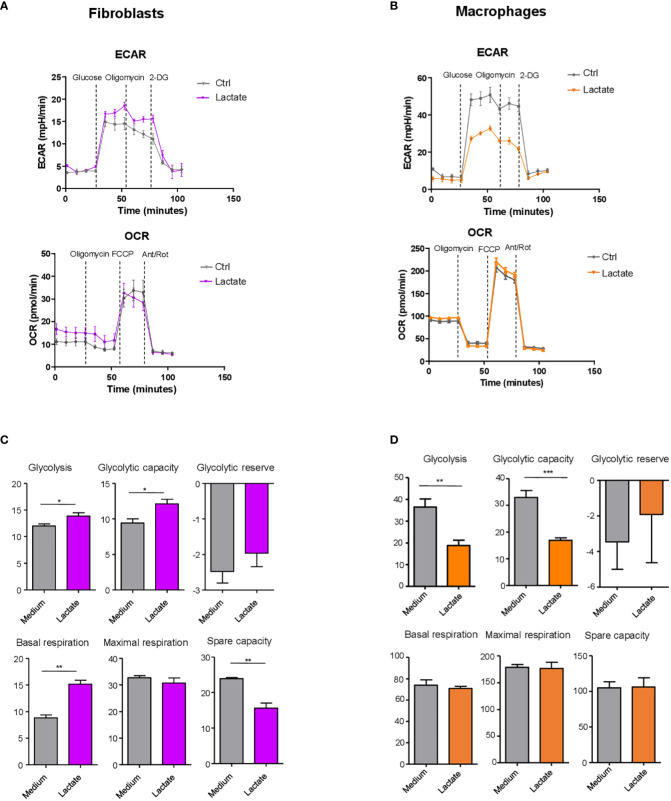
Lactate regulation of fibroblast and macrophage metabolism. **(A, B)** Seahorse analysis of extracellular acidification rate (ECAR, upper) and oxygen consumption rate (OCR, lower) in synovial fibroblasts (left) and monocyte-derived macrophages (right). **(C, D)** Glycolysis (after glucose injection), glycolytic capacity (after the injection of oligomycin) and glycolytic reserve (difference in ECAR between glucose and oligomycin injections) were calculated. Basal respiration [before oligomycin], maximal respiration [between FCCP and Antimycin+ rotenone injection], spare respiratory capacity [difference between basal and the maximal respiration], were calculated. Data expressed are representative of n=3 biological replicates. Data are expressed as mean ± SEM. Student’s t-test *p < 0.05; **p < 0.03; ***p < 0.01.

To directly interrogate the effect of lactate on fibroblasts and macrophages cellular metabolism (glycolysis and mitochondrial respiration), we used the Seahorse XF Analyzer in presence of compounds which inhibit or activate cellular bioenergetics (see figure legend, **Figure 2**). We sought to determine the capability of fibroblasts and macrophages to utilize these metabolic pathways under lactate treatment. This experiment was carried out on synovial fibroblasts cell lines derived from 3 different RA patients and monocyte-derived macrophages isolated from 3 healthy donors cultured in presence of M-CSF. ECAR and OCR were assessed as measures of aerobic glycolysis and mitochondrial respiration respectively (**Figures 2A, B**).

In fibroblasts, basal glycolysis and glycolytic capacity (maximal rate of glycolysis determined using the mitochondrial ATP synthase inhibitor oligomycin), were both increased in presence of lactate (**Figure 2C**). Mitochondrial parameters were measured in presence of oligomycin (ATP synthase inhibitor), FCCP (uncoupler of mitochondrial oxidative phosphorylation), and rotenone plus antimycin A to completely disrupt mitochondrial respiration. Basal respiration was significantly increased by lactate (**Figure 2C**). Maximal respiration did not differ between treatments. Spare respiratory capacity, known as the difference between maximal and basal respiration rates, was significantly reduced by lactate treatment (**Figure 2C**).

In contrast to fibroblasts, in macrophages ECAR (**Figure 2B**), basal glycolysis and glycolytic capacity (**Figure 2D**) were significantly reduced by lactate treatment, while respiratory parameters were not modulated by this metabolite (**Figure 2D**).

### 3.3 Differential effect of lactate on fibroblast and macrophage motility

Given the differential lactate-induced metabolic switch from resting conditions in fibroblasts and macrophages we asked whether lactate was able to promote differential effector functions, and in particular migration, in these two cell types.

**Figure 3:**
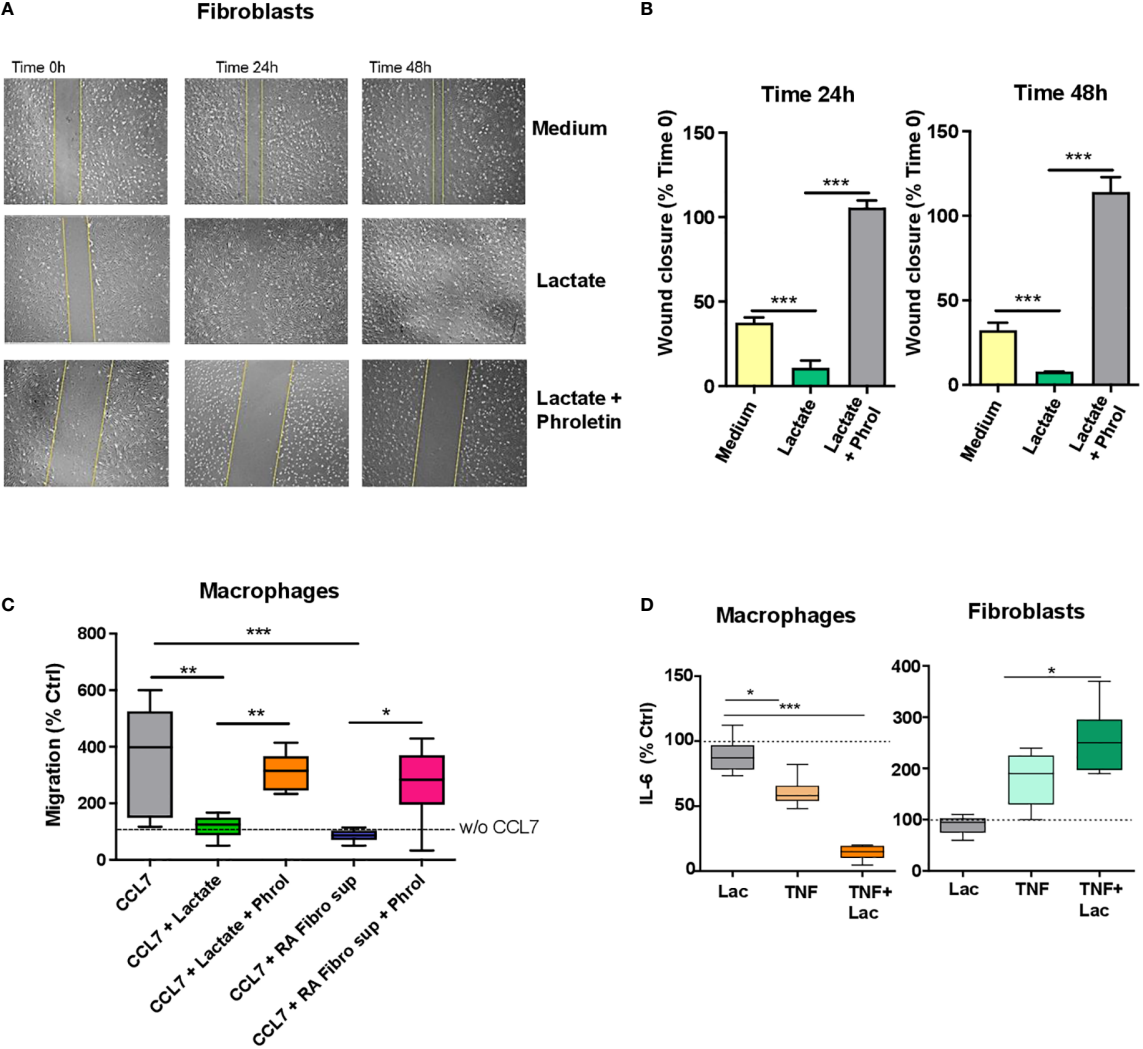
Lactate regulation of fibroblast and macrophage motility and IL-6 production. **(A)** Scratch test assay of synovial fibroblasts from RA patients (n=3 biological replicates). Cells were seeded for 24h and 48h in RPMI supplemented with 1% FBS and treated with or without sodium lactate (10 mM) +/- phroletin. **(B)** The perimeter (wound gap) of each scratch was measured with ImageJ software. The fold was calculated on Time 0 for each treatment (n=3, each in duplicate). **(C)** *In vitro* chemokinesis of monocytes-derived macrophages in response to CCL7 (300 ng/mL; n=6). Cells were cultured in medium containing sodium lactate (10 mM) or fibroblasts conditioned medium (10%) with or without phroletin (41 uM). Untreated macrophages (w/o CCL7, dotted line) were set to 100 (n=3, each in duplicate). **(D)** IL-6 ELISA from supernatants of fibroblasts and macrophages stimulated with TNFα (10 ng/ml) +/- lactate (n=3, each in duplicate). Untreated cells (dotted line) were set to 1. Data expressed are representative of n=3 biological replicates. Data are expressed as mean ± SEM. Anova test *p < 0.05; **p < 0.03; ***p < 0.01.

To study the effect of lactate on fibroblast migration, we used a scratch assay method, which is an *in vitro* assay to study cell migration (23). Cells were starved (1% FBS) and then treated with sodium lactate (10 mM) +/- pan-lactate transporter inhibitor phroletin (41 uM, 22) or left untreated. Images were acquired at time 0, 24h and 48h. Increased fibroblast migration was observed after 24h lactate treatment (**Figures 3A, B**); this was reversed by pre-treatment with lactate transporter inhibitor phroletin (**Figures 3A, B**).

To assess whether macrophage motility was affected by lactate or RA fibroblast supernatant, we performed a transwell assay whereby chemotaxis of unpolarized macrophages, was induced by the chemokine CCL7 which promotes chemotaxis of both M1 and M2 macrophages (24). Cells were treated with 10mM sodium lactate or RA fibroblast conditioned media +/- phroletin. Opposite to fibroblasts, macrophage motility was inhibited by sodium lactate or RA fibroblast conditioned medium. This effect was counteracted by pre-treatment with phroletin (**Figure 3C**). We didn’t observe increased cell death after treatment with lactate or phroletin at the above specified concentrations (data not shown).

### 3.4 Differential effect of lactate on fibroblast and macrophage IL-6 production

Fibroblasts and macrophages are the main source of IL-6 in arthritic joints (25, 26). To assess the effect of lactate on fibroblast and macrophage IL-6 production, synovial fibroblasts and monocyte-derived macrophages were cultured as described in Methods in presence of TNFα (10 ng/ml) +/- sodium lactate (10 mM). Lactate alone was unable to modulate IL-6 production while when in combination to TNF, lactate promoted an increase in IL-6 secretion in fibroblasts and a decrease in macrophages (**Figure 3D**).

### 3.5 Lactate exporter SLC16A3 correlates with histological and clinical score of disease

**Figure 4:**
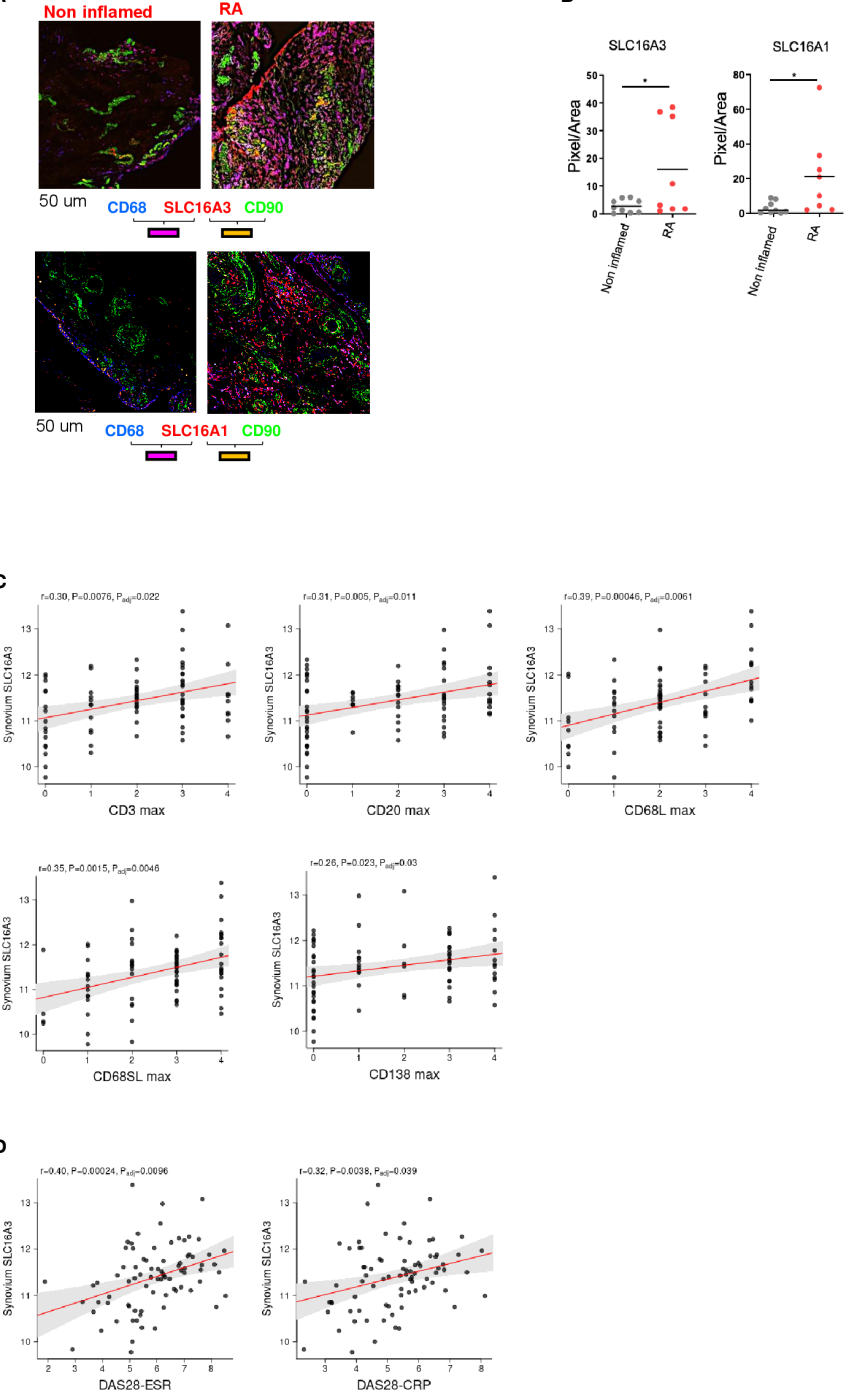
Correlation between lactate transporter expression and disease activity. **(A)** Representative images of SLC16A3 and SLC16A1 expression (red) by fibroblasts (CD90 green) and macrophages (CD68, blue) in RA and non-inflamed normal synovium. Scale bar 50 um. **(B)** Quantifications (pixel/area) of SLC16A3 and SLC16A1 expression in RA (n=8) and normal synovium (n=9). Data are expressed as mean ± SEM. Student’s t-test *p < 0.05. **(C)** Synovium SLC16A3 transcript positively correlates with histological score of synovitis (CD3, CD20, CD68 lining and sub lining, CD138) and with **(D)** the disease activity score (DAS28-ESR, DAS28-CRP) in a cohort of naïve-to treatment RA patients (27).

To investigate whether inflammation regulates the expression of these transporters, we compared the expression of SLC16A1 and SLC16A3 in RA (n=8) versus non inflamed tissue (n=9). We found that both transporters were upregulated in RA (**Figures 4A, B**).

We next correlated SLC16A1 and SLC16A3 synovial mRNA expression with histological and clinical parameters using a publicly available dataset of a cohort of adult, naïve to treatment RA patients (http://www.peac-mrc.mds.qmul.ac.uk/index.php) (27) with an arthritis duration <12 months. Synovial biopsies were classified according to the inflammatory score by histological analysis (28, 29). SLC16A3 mRNA expression positively correlated with the histological grade of synovitis (CD3, CD20, CD69 and CD138 infiltration) and the disease activity score (DAS28 ESR and CRP, **Figures 4C, D**). SLC16A1 did not show a similar correlation (**Supplementary Figure 1**).

## 4 Discussion

Dysregulated cellular metabolism is an important driver of chronic inflammation in several immune mediated inflammatory disorders such as RA (8).

Evidence accumulated during the past 15 years highlights an important role for metabolic pathways in the regulation of immune and stromal cell functions in RA joint. Indeed, several studies have demonstrated that a shift in bioenergetic profiles can shape the phenotype of a cell from a resting/resolving to a highly pro-inflammatory one (8).

Bioenergetic pathways, metabolic intermediates, transcription factors acting as metabolic sensors, are all required for an efficient and robust immune response and result in a rapid cellular metabolic reprogramming within the synovial tissue (8). As a result of this cellular turnover, the inflamed joint becomes a site of intense nutrient competition resulting in low levels of glucose and high amounts of lactate (30). Accumulation of lactate in RA synovium is in part the cause for its acidic pH which is significantly lower in inflamed RA joints than in healthy one (31). For many years, lactate has been considered a bystander product or a biomarker in sepsis and cancer. However, it has now been shown to be a major substrate for several metabolic pathways: for gluconeogenesis in the lactate-glucose cycle (Cori cycle), for oxidative phosphorylation in neurons (32), and for glycogen synthesis in the skeletal muscle (33, 34). Glycogen synthase has been shown to contribute to fibroblast-mediated synovial inflammation in RA (35). The mechanisms linking lactate, glycogen synthesis and inflammation in RA hasn’t been assessed so far.

In addition, lactate is a major carbon source for tricarboxylic acid (TCA) cycle in healthy and cancerous tissues. Indeed, infusion of labelled[U-13C]lactate in mice, led to accumulation of labelled TCA cycle intermediates in several tissues (36). Interestingly, in some tumours such as pancreatic and lung cancers, the supplying of lactate to the TCA cycle was much bigger than that of glucose (36). Moreover, lactate can shuttle between cells, tissues and organs (18) providing a flexible fuel for their metabolism. The stereoselective transport of lactate across cell membranes is mainly catalysed by monocarboxylate transporters (MCTs) part of the solute carrier (SLC)16 family (37, 38). SLC16A1 (MCT1) is ubiquitously expressed and regulates lactate-H^+^ import, while SLC16A3 (MCT4) which is strongly overexpressed in highly glycolytic and hypoxic tissues, is mainly responsible for lactate export (38); although the direction of transport is determined by the net driving force for lactate and H^+^. Normal functioning of lactate shuttle is important for homeostatic regulation; however, on some occasions this shuttling is maladaptive. For instance, in many cancer types, disease progression and aggressiveness are accompanied by hyperlactatemia (39). Lactate production and accumulation, phenomenon known as the “Warburg effect”, has gained enormous interest in the recent years in the cancer and inflammation field. Targeting lactate transporters has showed promising results in the inflammatory and cancer field (22, 38). Human and animal studies indicate they are druggable with SLC16A1 inhibitors being in advanced development phase and SLC16A3 inhibitors still in the discovery phase (22). By understanding and defining which specific metabolic requirements fuel a particular inflammatory immune disorder, new therapeutic targets might emerge. Little is known about how current therapeutic options for RA (e.g anti-TNF, anti-IL-6, anti-CD20) affect cellular metabolism including lactate metabolism. An interesting study showed that Tofacitinib (JAK/STAT inhibitor) significantly increased oxidative phosphorylation, ATP production, and the maximal respiratory capacity and the respiratory reserve in RA synovial fibroblasts, an effect paralleled by a decrease in glycolysis and glycolytic enzymes such as HK2, GSK-3α, lactate dehydrogenase A, HIF1α (40).

In this study we explored the hypothesis that synovial fibroblasts and macrophages, key drivers in RA pathogenesis (1–5), exchange lactate *via* the expression of distinct lactate transporters and utilize this metabolite for their effector functions and metabolism.

We started our analysis by showing the cellular expression of lactate transporters SLC16A1 and SLC16A3 which regulate lactate import and export respectively (22, 38), in RA synovium. Both transporters were expressed by synovial fibroblasts and macrophages, however macrophages mainly expressed SLC16A3, while fibroblasts favoured the expression of SLC16A1. This pattern of expression was maintained in both synovial compartments, namely lining and sub lining layers (**Figures 1A–D**).

Giving the different expression of lactate transporters, especially SLC16A3, on fibroblasts and macrophages we questioned whether lactate had a distinct effect on the metabolism and effector functions of these two cell types.

When stimulated with lactate we observed an increase of glycolysis and glycolytic capacity in synovial fibroblasts (**Figures 2A, C**) as previously reported for dermal fibroblasts (41) possibly due to reduced pyruvate dehydrogenase phosphorylation and oxidative phosphorylation (41). Similar to dermal fibroblasts we found an increase of basal respiration in synovial fibroblasts upon lactate stimulation; however, the spare respiratory capacity, was significantly reduced by lactate which is indicative of reduced response to metabolic stress (**Figures 2A, C**).

Conversely glycolysis and glycolytic capacity were significantly reduced in macrophages upon lactate treatment as previously described [(42), **Figures 2B, D**]. Indeed, when cells are exposed to high lactate levels, such as during inflammation, lactate dehydrogenase reaction is reversed with reduction of NAD^+^ to NADH and consequent inhibition of glycolysis (17).

It is well recognized that synovial fibroblasts acquire an invasive phenotype during the development of RA (43) which is in part responsible for joint destruction (43). We found that RA synovial fibroblasts when treated with sodium lactate increase their motility capabilities (**Figures 3A, B**); this effect was reversed by lactate transporter inhibition with phroletin suggesting that lactate-induced fibroblast motility is mediated by SLC16A1-3 (**Figure 3A, B**). In contrast to fibroblasts, macrophages responded to sodium lactate by inhibiting their motility (**Figure 3C**); again, this effect was reversed by the addition of phroletin in the cell culture (**Figure 3C**). A reduction of macrophage chemotaxis was also observed when cells were cultured in RA fibroblast conditioned media suggesting that lactate and other soluble factors produced by fibroblasts may regulate macrophage movement within the synovium. This is consistent with recent findings demonstrating that microenvironmental sensing by fibroblasts controls macrophage population size in a Hippo-YAP1 dependent mechanism (44). Since IL-6 is one of the most abundant pro-inflammatory cytokines found in the synovial fluid of RA patients (45), we investigated the effect of lactate on the production of IL-6 by resting or TNF-activated fibroblasts and macrophages (**Figure 3D**). Interestingly lactate was able to increase or abrogate IL-6 production by TNF-stimulated fibroblasts and macrophages respectively; suggesting that these two cell types utilise distinct metabolic machineries for IL-6 production (**Figure 3D**). How metabolism regulates IL-6 production needs further investigation; however, one possibility might be epigenetic lactate regulation (46). It has been previously shown that lactate promotes HIF1α activation with a consequent metabolic reprogramming in fibroblasts (41). In addition, once activated, HIF1α promotes the expression of several proinflammatory cytokines including IL-6 (47). Thus, it’s possible to speculate that lactate regulation of IL-6 production is mediated by HIF however this would need further direct validation. Lactate itself was unable to regulate Il-6 secretion from synovial fibroblasts without TNF (**Figure 3D**). One possible explanation is that fibroblasts need to be primed (activated) by one or more inflammatory stimuli such as TNF in addition to lactate in order to promote chronic inflammation (48) and as we have previously showed for T cells (17).

Overall, these results support the hypothesis that macrophages and fibroblasts use lactate for different purposes. Macrophages appear to be more reliant on glycolytic metabolism and respond to high level of lactate by upregulating SLC16A3 in order to prevent intracellular lactic acidosis and cell death (49, 50).

Several evidence has identified SLC16A3 highly expressed by monocyte-derived macrophages isolated from healthy volunteers’ peripheral blood. This expression is further up-regulated by toll-like receptors (TLR)2 and TLR4 agonists in a NF-κB-dependent manner (49). Moreover, pharmacological or genetic (siRNA) inhibition of SLC16A3 reduced the levels of pro-inflammatory molecules including IL-1β, IL-6, TNFα, and iNOS in LPS-stimulated macrophages (M1 phenotype) attenuating inflammation. Macrophages also express SLC16A1 (MCT1) through which they can uptake lactate, in an autocrine or paracrine way, which in turn promotes their differentiation in a regulatory anti-inflammatory phenotype (M2 phenotype) (50, 51). In our study we found that exposure of macrophages to lactate is accompanied by a reduction of glycolysis, migration and IL-6 production. This is consistent with previous evidence showing lactate mediated polarization of macrophages toward a M2 phenotype (52). Fibroblasts on the other hand likely uptake lactate *via* SLC16A1 fuelling their combined oxidative and glycolytic metabolism. When exposed to lactate fibroblasts acquire a pro-inflammatory phenotype by increasing their migration and IL-6 secretion.

The divergent effects of lactate on macrophages and fibroblasts suggest that lactate may represent a key metabolite regulating the choreography between these two cell types in the synovium which we hypothesize becomes dysfunctional in disease.

Finally, we found that both SLC16A1 and SLC16A3 were more expressed in RA in comparison to non-inflamed synovium (**Figures 4A, B**) and SLC16A3 but not SLC16A1 directly correlated with the clinical and histological disease state using a publicly available RNAseq dataset (27). Taken together these data suggest that lactate and its transporters may be potential targets for future therapeutic option in RA and other inflammatory disorders where fibroblast and macrophage metabolic cross talk plays a role.

## Data availability statement

The raw data supporting the conclusions of this article will be made available by the authors, without undue reservation.

## Ethics statement

The studies involving human participants were reviewed and approved by West Midlands - Black Country Research Ethics Committee (12/WM/0258). The patients/participants provided their written informed consent to participate in this study.

## Author contributions

Conceptualisation, VP, GA, CB, KR, AC. Methodology, VP, MN, VG, SC, JM. Investigation VP, MN, VG, SC, JM. Analysis, VP, MN, VG, JM. Resources, AF, CB, KR, AC. Writing – Original Draft, VP, GA, KR, and CB. Writing – Review and Editing, all authors. Visualisation, VP, KR, and CB. Supervision, AF, AC, CB, KR, AR.
