# Supplementary figures and images for: Differential effect of lactate on synovial fibroblast and macrophage effector functions
Source: Front Immunol. 2023 May 22;14:1183825. doi: 10.3389/fimmu.2023.1183825 (PMC10251493; doi:10.3389/fimmu.2023.1183825)

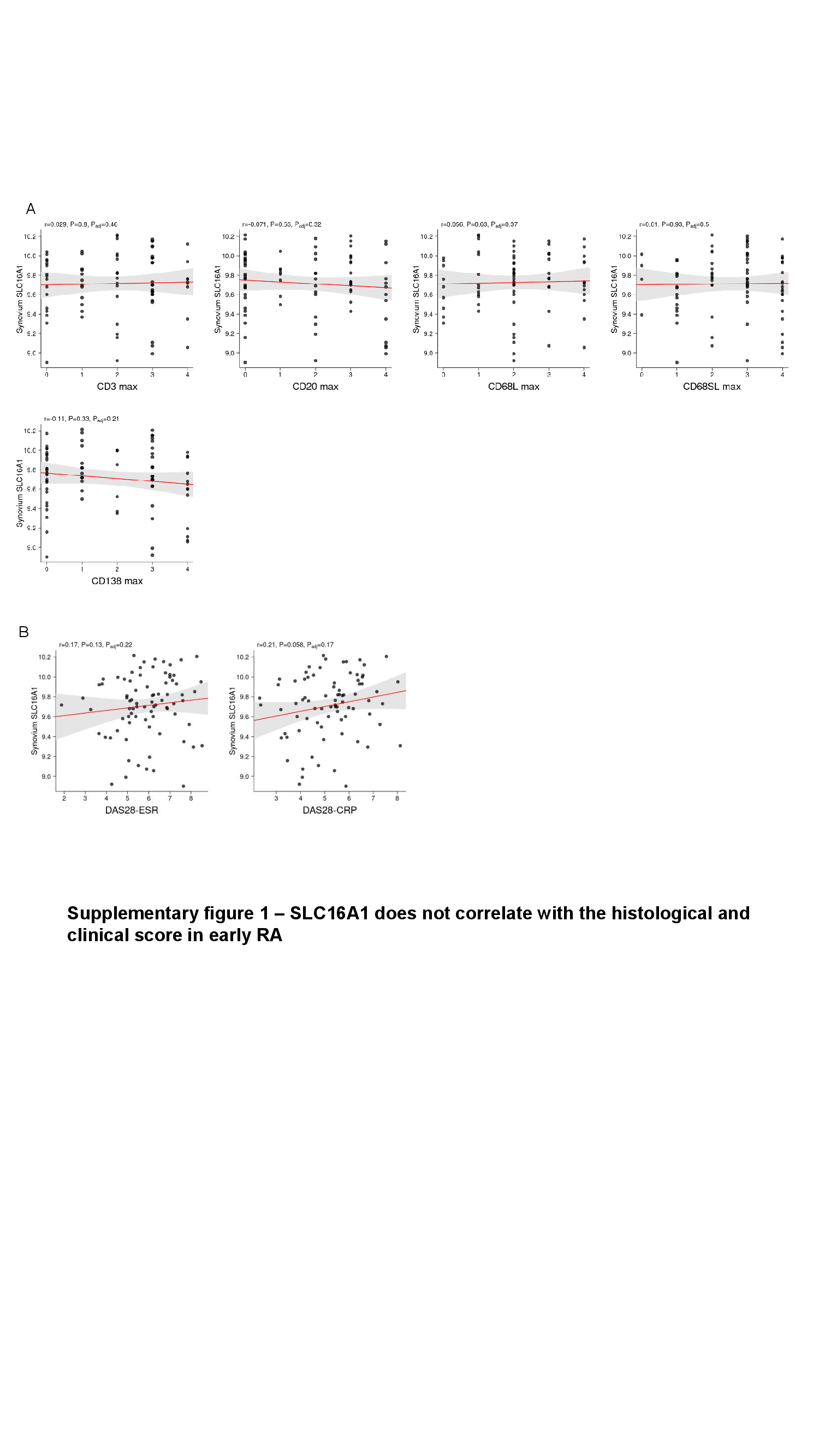

Supplement: Supplementary file 1 [file Image_1.tiff]
